# Cerebrovascular Manifestations of Lyme Neuroborreliosis—A Systematic Review of Published Cases

**DOI:** 10.3389/fneur.2017.00146

**Published:** 2017-04-20

**Authors:** Adam Garkowski, Joanna Zajkowska, Agata Zajkowska, Alina Kułakowska, Olga Zajkowska, Bożena Kubas, Dorota Jurgilewicz, Marcin Hładuński, Urszula Łebkowska

**Affiliations:** ^1^Department of Radiology, Medical University of Białystok, Białystok, Poland; ^2^Department of Infectious Diseases and Neuroinfections, Medical University of Białystok, Białystok, Poland; ^3^Department of Neurology, Medical University of Białystok, Białystok, Poland; ^4^Faculty of Applied Informatics and Mathematics, Warsaw University of Life Sciences SGGW, Warsaw, Poland; ^5^Independent Department, Laboratory of Molecular Imaging, Medical University of Białystok, Białystok, Poland

**Keywords:** Lyme neuroborreliosis, stroke, vasculitis, vasculopathy, *Borrelia burgdorferi*, cerebrovascular manifestations

## Abstract

**Background:**

Lyme neuroborreliosis (LNB) is a disease caused by spirochete *Borrelia burgdorferi*, involving the nervous system. It usually manifests as lymphocytic meningoradiculitis, but in rare cases, it can also lead to cerebrovascular complications. We aimed to perform a systematic review of all reported cases of LNB complicated by central nervous system vasculitis and stroke or transient ischemic attack (TIA).

**Materials and methods:**

We conducted a systematic review of literature between May 1987 and December 2016 with patients who presented with cerebrovascular course of LNB.

**Results:**

This study included 88 patients with a median age of 46 years. The median interval from onset of symptoms suggesting Lyme disease to first symptoms of cerebrovascular manifestations of LNB was 3.5 months. The most common cerebrovascular manifestation of LNB was ischemic stroke (76.1%), followed by TIA (11.4%). The posterior circulation was affected alone in 37.8% of patients, the anterior circulation in 24.4% of patients, and in 37.8% of cases, posterior and anterior circulations were affected simultaneously. The most common affected vessels were middle cerebral artery—in 19 cases, basilar artery—in 17 cases, and anterior cerebral artery—in 16 cases. A good response to antibiotic treatment was achieved in the vast number of patients (75.3%). The overall mortality rate was 4.7%.

**Conclusion:**

Cerebral vasculitis and stroke due to LNB should be considered, especially in patients who live in or have come from areas with high prevalence of tick-borne diseases, as well as in those without cardiovascular risk factors, but with stroke-like symptoms of unknown cause.

## Introduction

Lyme disease, multisystem and multistage infectious disease, is the most common tick-borne disease in Europe and North America and is caused by infection with spirochetes of the *Borrelia burgdorferi* sensu lato genospecies complex. The different genospecies are associated with distinct clinical manifestations. Lyme neuroborreliosis (LNB) is more common in Europe than in the United States and typically presents as Bannwarth’s syndrome including lymphocytic meningitis, cranial neuritis, and radiculoneuritis. These manifestations can occur separately or together (1, 2).

In most cases, cerebrovascular events are attributed to arteriosclerosis or cerebral embolism, but sometimes infectious causes are found. *B. burgdorferi*-induced CNS vasculitis is believed to be a very rare manifestation of LNB and may manifests as ischemic stroke (3), transient ischemic attack (TIA) (4), and very rarely as intracranial hemorrhage (ICH) (5, 6), cerebral venous sinus thrombosis (CVST) (7, 8), or aneurysm (9, 10).

To date, there has been no comprehensive systematic review of the literature on cerebrovascular manifestations of LNB with the aim of clarifying the epidemiology, clinical course, imaging studies, and the outcome of these rare manifestations of LNB. We aimed to collect all the articles with patients who presented with vasculitis and stroke due to LNB and systematically review the data reported.

## Materials and Methods

We conducted a systematic review of the medical literature to identify all published cases of cerebrovascular manifestations of LNB using the online databases of MEDLINE/PubMed, Web of Science and Google Scholar (between May 1987 and December 2016). There were no language restrictions; non-English language articles were included and translated. The search terms were “Lyme disease” OR “neuroborreliosis” OR “borreliosis” OR “*Borrelia burgdorferi*” AND one of the following terms: “vasculitis” OR “vasculopathy” OR “angiopathy” OR “stroke” OR “transient ischemic attack” OR “intracranial haemorrhage” OR “subarachnoid hemorrhage” OR “venous thrombosis.” We reviewed titles, abstracts, and full articles to assess if our inclusion criteria had been met. References in each identified articles were reviewed to identify additional cases that we had not found by using the search terms. In addition, the author, date, city, and hospital of each case report were verified for duplication of reporting. The review followed the Preferred Reporting Items for Systematic Reviews and Meta-Analyses guidelines.

Our inclusion criteria included (1) clinical and/or radiologic evidence of cerebrovascular manifestations of LNB (vasculitis, stroke, TIA, or aneurysms), (2) mandatory confirmation of LNB according to the European Federation of Neurological Societies (EFNS) guidelines (definite or possible) (11) or guidelines of the Infectious Diseases Society of America (12) and the American Academy of Neurology (13), and (3) excluded other stroke risk factors (e.g., atrial fibrillation or infective endocarditis, neurosyphilis, and other infectious causes).

Information from each article was extracted and entered into Microsoft Excel (2007). We extracted the following data from each article: age, sex, country of origin, history of tick bite, preceding symptoms of Lyme disease, time from onset of symptoms suggestive of Lyme disease to onset of symptoms of stroke (or TIA), neurological symptoms at presentation, the CSF cells, protein and glucose levels, imaging studies performed with evaluation of distribution of lesions, treatment, and outcome. Stroke was defined according to the World Health Organization as “rapidly developing clinical signs of focal (or global) disturbance of cerebral function, with symptoms lasting 24 h or longer or leading to death, with no apparent cause other than of vascular origin.” TIA was defined as a transient episode of cerebral dysfunction lasting less than 24 h (14).

Based on vascular studies, which were available in most cases, we classified LNB-associated vasculitis into three main groups: (1) large-sized blood vessels vasculitis; (2) small-sized blood vessels vasculitis, or (3) variable-sized blood vessels vasculitis (both large- and small-sized vessels). Large vessels included internal carotid arteries (ICA), the vertebral arteries (VA), the anterior cerebral artery (ACA) and their main branches, the middle cerebral arteries (MCA) and their main branches, the posterior cerebral arteries (PCA), the basilar artery (BA) and their main branches, the transverse sinuses, the sigmoid sinuses, and the superior sagittal sinus. Small vessels included the small penetrating arteries (e.g., the lenticulostriate arteries) that supplied the deep structures of the brain.

On account of the fact that this was a review of published literature, the ethics committee approval was not required. Statistical analysis was performed using the STATISTICA 10 software.

## Results

**Figure 1:**
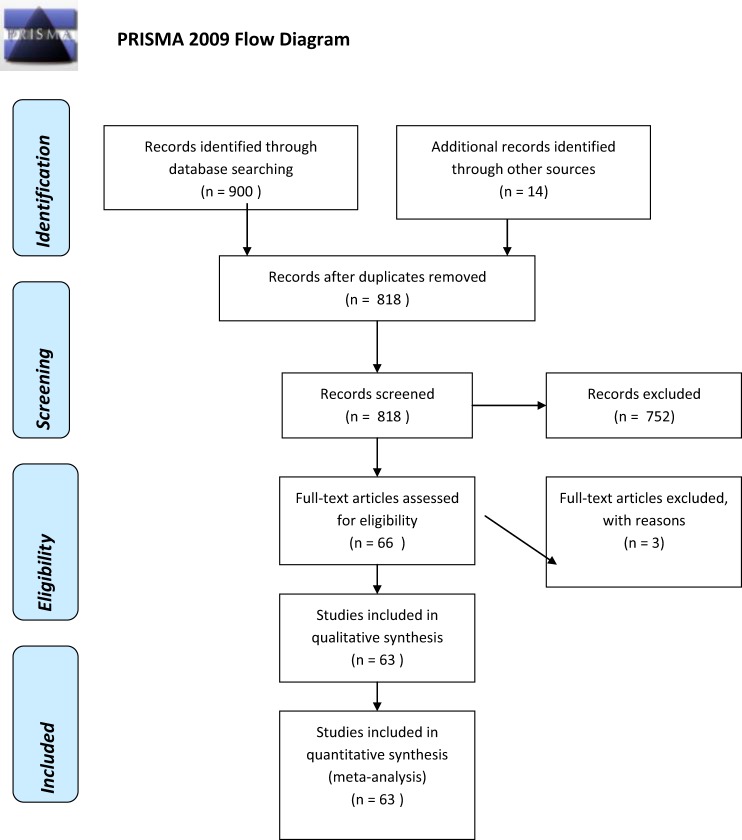
**Preferred Reporting Items for Systematic Reviews and Meta-Analyses (PRISMA) flowchart illustrating the number of included and excluded studies in the systematic review on cerebrovascular manifestations of Lyme neuroborreliosis**.

After a strict selection process, a total of 63 manuscripts fulfilled the inclusion criteria and were reviewed in this study (3–10, 15–70) (Figure 1). We excluded four patients from three manuscripts because they did not meet the criteria for LNB (9, 37, 71). Finally, we analyzed 88 individual cases from these manuscripts. The median age of the patients at the time of diagnosis was 46 (range from 4 to 77 years), 47 patients (53.4%) were male; 52 (59.1%) of patients were younger than 50 years. There were 15 pediatric cases. Most patients were from countries with high incidence of reported Lyme disease (72). Reports from the following three countries account for more then the 50% of the included cases: 16 (25.4%) from Germany, 9 (14.3%) from Switzerland, and 8 (12.7%) from France. The clinical and epidemiological characteristics of patients are shown in Tables 1 and 2. Data Sheet S1 in the Supplementary Material presents detailed clinical features, CSF cytosis, and imaging studies of all reported cases.

**Table 1:** **Epidemiological characteristics of patients with cerebrovascular manifestations of Lyme neuroborreliosis**.

	*n*/*N* (%)
**Characteristic**	
Median age, years	46 (4–77)
Male sex	47/88 (53.4%)
**Origin of the manuscripts**	
Germany	16/63 (25.4%)
Switzerland	9/63 (14.3%)
France	8/63 (12.7%)
United States	5/63 (8%)
Netherlands	5/63 (8%)
Sweden	4/63 (6.3%)
Croatia	3/63 (4.8%)
Austria	3/63 (4.8%)
Norway	2/63 (3.2%)
Finland	2/63 (3.2%)
Belgium	2/63 (3.2%)
Poland	1/63 (1.6%)
Spain	1/63 (1.6%)
Turkey	1/63 (1.6%)
United Kingdom	1/63 (1.6%)
**Response to treatment**	
Complete	64/85 (75.3%)
Incomplete/stable with residual neurological deficits	17/85 (20%)
Fatal	4/85 (4.7%)

*n/N (%)—n is the number of patients in which the variable was present and N the total number of patients for which that particular variable was reported*.

**Table 2:** **Clinical characteristics of patients with cerebrovascular manifestations of Lyme neuroborreliosis (LNB)**.

Prodromal clinical course	*n*/*N* (%)
History of tick bite	40/68 (58.8%)
History of erythema migrans	15/57 (26.3%)
History of headache	41/65 (63.1%)
History of radiculitis	20/58 (34.5%)
History of cranial neuritis	18/59 (31%)
History of arthritis	5/56 (9%)
**CSF analysis**	
Median cytosis	77.5 cells/μL (range 1–2.200)
Median protein level	1.400 mg/L (range 162–7.458)
Median glucose level	1.7 mmol/L (range 0.7–4.2)
**Type of cerebrovascular manifestation of LNB**	
Ischemic stroke	67/88 (76.1%)
Transient ischemic attack	10/88 (11.4%)
Cerebral venous sinus thrombosis (CVST)	3/88 (3.4%)
Intracranial hemorrhage	3/88 (3.4%)
SAH	1/88 (1.1%)
Ischemic stroke + CVST	1/88 (1.1%)
Ischemic stroke + aneurysm	1/88 (1.1%)
SAH + aneurysm	1/88 (1.1%)
**Vascular studies (magnetic resonance angiography, computed tomography angiography, DSA, transcranial Doppler)**	**66/88 (75%)**
**Affected vessels**	
Large-sized	42/66 (63.6%)
Small-sized	9/66 (13.6%)
Variable-sized	1/66 (1.5%)
**Affected areas of circulation**	
Posterior	31/82 (37.8%)
Anterior	20/82 (24.4%)
Anterior + posterior	31/82 (37.8%)
**Distribution of lesions on CT and/or MRI**	
Single	25/88 (28.4%)
Multiple	50/88 (56.8%)
No lesions	13/88 (14.8%)

*n/N (%)—n is the number of patients in which the variable was present and N the total number of patients for which that particular variable was reported*.

About 40 (58.8%) of patients had a history of tick bite, and about 15 (26.3%) of patients had a history of preceding erythema migrans. Forty-one (63.1%) of patients reported a history of headache, 20 (34.5%) of patients had a history of radiculitis, and 18 (31%) had a history of cranial neuritis. About five (9%) of patients had a history of arthritis. The median interval from symptom onset suggesting Lyme disease to first cerebrovascular manifestation of LNB was 3.5 months (range 0–48 months). In about 40.3% of patients, cerebrovascular LNB was the first manifestation. In 54 (61.4%) of patients, the symptoms occurred single and in 33 (37.5%) of cases were recurrent before the diagnosis of cerebrovascular LNB was made and the antibiotic treatment was commenced.

The most common cerebrovascular manifestation of LNB was ischemic stroke—67 cases (76.1%), followed by TIA—10 cases (11.4%). The rarest manifestations were CVST—three cases (3.4%), ICH—three cases (3.4%), SAH—three cases (1.1%), one case of ischemic stroke coexisting with CVST (1.1%) and with intracranial aneurysms in another case (1.1%), as well as one case of coexistence of SAH with intracranial aneurysm (1.1%). As far as available, in patients whose imaging examination showed pathological changes, it was alleged that the posterior cerebral circulation was affected alone in 31 patients (37.8%), while the anterior circulation was affected alone in 20 patients (24.4%). The anterior and posterior circulations were simultaneously affected in 31 cases (37.8%). All patients underwent MRI and/or CT of the brain. Among them, 75 patients (85.2%) had parenchymal brain lesions diagnosed at imaging; in the remaining patients, there were no parenchymal brain lesions. These lesions were multiple in 50 patients (66.7%) and single in 25 patients (33.3%). In 9 of 13 patients who did not show any parenchymal lesions on MRI and/or CT, cerebral angiography and/or transcranial Doppler (TCD) were performed simultaneously, visualizing the changes suggestive of vasculitis in all these cases; 4 of these 13 cases had performed neither TCD nor cerebral angiography. Among 27 patients who had simultaneously performed MRI and CT of the brain, parenchymal brain lesions were seen only on MRI in 41% of patients and were not seen on CT. Among patients for whom data on MRI meningeal enhancement were available, it was seen only in 13 patients. According to available data on vascular studies, among 66 patients (75%) who underwent such studies [conventional angiography, magnetic resonance angiography (MRA), computed tomography angiography (CTA), or TCD], 52 cases (78.8%) showed vascular abnormalities suggestive of vasculitis. Vascular studies showed large-sized blood vessels vasculitis in 63.6% of patients and variable-sized blood vessels vasculitis in 13.6% of patients followed by exclusively small-sized blood vessels vasculitis only in one case. The most common affected vessels (bilaterally or unilaterally) were MCA—in 19 cases, BA—in 17 cases, ACA—in 16 cases, PCA—in 9 cases, VA—in 8 cases, intracranial part of ICA—in 7 cases, and the penetrating arteries supplying the deep-seated structures—in 5 cases.

All patients underwent lumbar puncture as part of the diagnostic workup, and among the patients in whom CSF results were reported in the manuscripts, 96.2% of them had lymphocytic pleocytosis and 90% had increased protein level. The median CSF cytosis was 77.5 cells/μL (range 1–2.200), and the median CSF protein concentration was 1.400 mg/L (range 162–7.458). Among patients for whom data on CSF glucose level were available, 74% of them had decreased level of glucose [the median CSF glucose level was 1.7 mmol/L (range 0.7–4.2)]. Four patients had biopsy-proven CNS vasculitis. In the remaining patients, biopsy and histopathology of the brain were not performed.

The majority of patients were treated with ceftriaxone or penicillin. Data on additional treatment with steroids were available for 55 patients, wherein at least 25 of them (45.5%) received steroids, either *per os* or intravenously. Considering the reports wherein, information about antiplatelet and/or anticoagulation therapy was available, about 86.8% of the patients received such treatment. In three patients clinical improvement was achieved only after additional immunosuppressive therapy with cyclophosphamide.

Data on outcome were available in 85 cases. A complete response to antibiotic treatment with halted progression of the disease and no recurrence of cerebral ischemia or recuperation from neurological deficits was achieved in the vast number of patients (75.3%). The overall mortality rate was 4.7%. Among patients who died, posterior circulation was affected in two cases, and multiple vascular territories were affected in two cases.

## Discussion

In this systematic review of the literature, we have analyzed a large number of cases of CNS vasculitis and stroke associated with infection with *B. burgdorferi*, published as single case reports or small series. Although no gender predisposition was observed, the present study shows that patient with LNB-associated vasculitis are relatively young (median age 46 vs. 73 years in the one population-based stroke register from Europe) (73). However, this is probably due to the fact that the diagnostics for the causes of vasculitis and stroke is studied more accurately in younger patients. LNB seems to be a rare cause of vasculitis and stroke. In the study by Hammers-Berggren et al., including 281 patients with a diagnosis of stroke or TIA, 8% of them had positive serology for *B. burgdorferi* infection, but only one of them had stroke-like symptoms due to LNB. It suggests that screening for *B. burgdorferi* infection is not indicated in all patients with stroke (27). In the study by Back et al., the estimated frequency of cerebral vasculitis in patients with Lyme borreliosis amounted to 0.3% (57). In another study of 330 patients with LNB from Bavaria and Hesse (Germany) conducted by Oschmann et al., this variant has been seen in 1% of the patients (74). In typical cases of LNB-associated vasculitis, the patients live in an endemic area and have a medical history of tick bite, erythema migrans, headache, radiculitis, and/or cranial neuritis. The patients may also report non-specific complaints, such as fatigue or difficulty concentrating. Thus, it is important to take a detailed medical history from the patients. The case reports came predominantly from Europe, while only five cases were reported from the United States, which is related to the presence of *Borrelia garinii* in Europe, which is the most neurotropic genospecies of *Borrelia*. It should be noted that one of these five patients was infected with *B. burgdorferi* in Europe and diagnosed in the United States (23).

Parenchymal brain lesions suggestive of ischemia were present in the majority of patients, and in 66.7% of cases, they were multiple, which is typical for CNS vasculitis. MRI is the most sensitive imaging technique for detection of parenchymal changes associated with LNB vasculitis. In LNB, MRI may show meningeal enhancement after contrast administration; however, it is rarely reported. In this study, it was seen only in a small number of patients. Diagnosis of LNB-associated CNS vasculitis is primarily based on radiological investigations such as MRI and MRA or conventional angiography. As shown by the results, LNB-associated vasculitis affects mainly the large/medium-sized vessels causing single or multiple areas of stenosis and dilatation and as the end result occlusion leading to ischemia and stroke. In some patients presented with stroke, angiographic showed no features of vasculitis. Normal angiography may be due to the delay between the stroke symptoms onset and the radiological examination. Moreover, it is important to bear in mind that LNB-associated vasculitis may also involve small blood vessels, which are below the resolution of conventional angiography. MRA and CTA have even less sensitivity and most of the reported cases had performed such type of angiographic procedures. Therefore, a negative angiogram does not exclude the diagnosis of vasculitis (75).

CVST, ICH, SAH, and aneurysms were the rarest cerebrovascular complications. There is known association of another spirochete, *Treponema pallidum* causing syphilis, to aneurysm formation, indicates that there might be a causative relationship between Lyme disease, similar to syphilis in different stages of the disease, and aneurysm formation (76, 77). It is worth pointing out that a recent study on a group of 96 patients diagnosed with abdominal aortic aneurysms points out to a link between infection with *B. burgdorferi* and abdominal aortic aneurysms (78).

According to the literature, infection of CNS with *B. burgdorferi* typically leads to lymphocytic pleocytosis and increased CSF protein concentration (79, 80). In this study, CSF analysis demonstrated low median glucose level, which is atypical for LNB, wherein glucose level is usually normal. The combination of lymphocytic pleocytosis, elevated protein, and hypoglycorrhachia resembles tuberculous meningitis.

When a clinical diagnosis of LNB-associated vasculitis is suspected and supported by lesions on MRI or CT, further diagnostic testing should be performed, including CSF analysis and *B. burgdorferi* antibody testing of serum and CSF (2). To prove *B. burgdorferi*-associated CNS involvement, demonstration of *B. burgdorferi* intrathecal antibody production is used by calculating the CSF to serum antibody index (AI). The anti-*Borrelia* AI have about 80% sensitivity in LNB of short duration (<6 weeks) and almost 100% sensitivity within a few months and in late LNB. In the case of negative AI, the diagnosis of LNB may be made if there are positive *B. burgdorferi* antibodies in the serum and symptoms suggestive of CNS involvement. A positive AI may persist for years after successful therapy (11).

In our review, only a small number of cases had brain biopsy. It should be emphasized that only brain biopsy can provide a definite diagnosis of CNS vasculitis, but because it is an invasive diagnostic procedure, it is barely performed.

As can be seen from the results, LNB-induced vasculitis is highly responsive to appropriate antimicrobial treatment similarly to other forms of Lyme disease, but it is obvious that in some cases, especially in those where antibiotic therapy was delayed, permanent neurological deficits caused by CNS injury and scarring that had occurred prior to the treatment can occur. Vasculitis in the posterior circulation was associated with worse prognosis. According to the current EFNS guidelines, adult patients with early LNB with CNS manifestations (including vasculitis) should be treated with intravenous (IV) ceftriaxone (2 g daily) for 14 days. The patients with late LNB with CNS manifestations (also including vasculitis) should be treated with IV ceftriaxone (2 g daily) for 3 weeks (11). Nonetheless, it should be noted that a recent study has indicated that oral doxycycline is as effective treatment for LNB as IV ceftriaxone (81). In the vast majority of patients, a significant improvement was obtained after antibiotics administration, indicating a direct vascular injury by *B. burgdorferi*. Many authors additionally used steroids. However, in some reported cases, clinical improvement was achieved only after additional immunosuppressive therapy with cyclophosphamide suggesting the additional role of immune mechanisms in the development of vasculitis.

Among fatal cases, one patient died in the acute stage due to extensive BA thrombosis (57). The other patient presented with an infarction in the territory of the left ACA and extensive changes in the BA and both MCAs, which subsequently evolved into cerebral edema leading to fatal transtentorial and subfalcine herniation (51). Another patient was admitted to hospital because of acute right-sided hemiparesis progressing to tetraparesis and respiratory failure in few hours. Cranial CT showed diffuse cerebral edema and MRI of the brain revealed multiple hyperintense lesions. The patient died a few days after being admitted to hospital due to cardiac arrest (60). The last patient who died developed severe hemiparesis and respiratory failure. The cause of death was bilateral pneumonia. In this patient, an autopsy was performed, which revealed infarcts in tegmentum and medulla oblongata. There was no evidence of atherosclerosis in the vascular lumen; however, the clear evidence of CNS vasculitis was observed (26).

There are some limitations to our study that are inherent to any systematic review. Limits in our results are due to incomplete information in the case descriptions on all variables, especially data on the history of preceding symptoms of Lyme disease or information about time from onset of symptoms suggestive of Lyme disease to onset of symptoms of cerebrovascular complications of LNB. Furthermore, data on additional steroids and antiplatelet drugs were only known for 55 and 38 patients, respectively. In three patients, there was a lack of outcome data. Although we have created a literature search algorithm to identify all cases of CNS vasculitis due to LNB, we cannot exclude an involuntary omission. However, despite these limitations, our review summarizes the epidemiology, clinical manifestations, neuroimaging, and outcomes in patients presenting with cerebrovascular manifestations of LNB.

## Conclusion

It is concluded that patients with LNB-associated vasculitis usually achieve a favorable recovery. As a rare cause of stroke or TIA, cerebral vasculitis due to LNB should be considered. Several weeks/months before stroke onset, prodromal symptoms suggesting LNB, such as meningitis, cranial neuritis or radiculoneuritis, should prompt extensive diagnostics including CSF examination. Due to the fact that it can appear even in the absence of earlier more characteristic symptoms of Lyme disease, LNB-associated vasculitis should be excluded in all patients with vasculitis and multiple ischemic lesions of unknown origin, especially in those living in endemic areas. The lack of awareness of this manifestation of LNB might result in the delay of diagnosis, which might lead even to the patient’s death. Perhaps, this presentation of LNB may occur more frequently than it is thought. In patients suspected of having *B. burgdorferi*-induced CNS vasculitis and stroke, the most useful and crucial imaging examination to evaluate cerebral vessels is cerebral angiography (conventional angiography, MRA, or CTA), which may reveal focal narrowing and dilatation or even total occlusion of the arteries.

## Author Contributions

The conceiving and the conception of research were contributed by AG. The search strategies were designed by AG, JZ, AZ, and AK. The data were analyzed and interpreted and the final version of the manuscript was read and approved by AG, JZ, AZ, AK, OZ, BK, DJ, MH, and UŁ. The paper was written by AG, JZ, AZ, and UŁ.

## Conflict of Interest Statement

The authors declare that the research was conducted in the absence of any commercial or financial relationships that could be construed as a potential conflict of interest. The reviewer, NM, and handling editor declared their shared affiliation, and the handling editor states that the process nevertheless met the standards of a fair and objective review.

## References

[1] Stanek G Fingerle V Hunfeld KP Jaulhac B Kaiser R Krause A Lyme borreliosis: clinical case definitions for diagnosis and management in Europe. Clin Microbiol Infect (2011) 17:69–79. 10.1111/j.1469-0691.2010.03175.x 20132258

[2] Koedel U Fingerle V Pfister HW. Lyme neuroborreliosis-epidemiology, diagnosis and management. Nat Rev Neurol (2015) 11:446–56. 10.1038/nrneurol.2015.121 26215621

[3] Uldry PA Regli F Bogousslavsky J Cerebral angiopathy and recurrent strokes following *Borrelia burgdorferi* infection. J Neurol Neurosurg Psychiatry (1987) 50:1703–4. 10.1136/jnnp.50.12.1703 3437311 PMC1032623

[4] Midgard R Hofstad H. Unusual manifestations of nervous system *Borrelia burgdorferi* infection. Arch Neurol (1987) 44:781–3. 10.1001/archneur.1987.00520190085021 3036051

[5] Seijo Martínez M Grandes Ibáñez J Sánchez Herrero J García-Moncó JC. Spontaneous brain hemorrhage associated with Lyme neuroborreliosis. Neurologia (2001) 16:43–5. 11234662

[6] Scheid R Hund-Georgiadis M von Cramon DY. Intracerebral haemorrhage as a manifestation of Lyme neuroborreliosis? Eur J Neurol (2003) 10:99–101. 10.1046/j.1468-1331.2003.00541.x 12535004

[7] Adamaszek M Heinrich A Rang A Langner S Khaw AV Cerebral sinuvenous thrombosis associated with Lyme neuroborreliosis. J Neurol (2010) 257:481–3. 10.1007/s00415-009-5397-7 19941147

[8] Ansari I Crichlow B Gunton KB Diamond GR Melvin J A child with venous sinus thrombosis with initial examination findings of pseudotumor syndrome. Arch Ophthalmol (2002) 120:867–9. 12049606

[9] Oksi J Kalimo H Marttila RJ Marjamäki M Sonninen P Nikoskelainen J Intracranial aneurysms in three patients with disseminated Lyme borreliosis: cause or chance association? J Neurol Neurosurg Psychiatry (1998) 64:636–42. 10.1136/jnnp.64.5.636 9598680 PMC2170070

[10] Polet JD Weinstein HC Lyme borreliosis and intracranial aneurysm. J Neurol Neurosurg Psychiatry (1999) 66:806–7. 10.1136/jnnp.66.6.806a PMC1736382 10400516

[11] Mygland A Ljøstad U Fingerle V Rupprecht T Schmutzhard E Steiner I European Federation of Neurological Societies: EFNS guidelines on the diagnosis and management of European Lyme neuroborreliosis. Eur J Neurol (2010) 17:8–16. 10.1111/j.1468-1331.2009.02862.x 19930447

[12] Wormser GP Dattwyler RJ Shapiro ED Halperin JJ Steere AC Klempner MS The clinical assessment, treatment, and prevention of Lyme disease, human granulocytic anaplasmosis, and babesiosis: clinical practice guidelines by the Infectious Diseases Society of America. Clin Infect Dis (2006) 43(9):1089–134. 10.1086/508667 17029130

[13] Halperin JJ Shapiro ED Logigian E Belman AL Dotevall L Wormser GP Practice parameter: treatment of nervous system Lyme disease (an evidence-based review): report of the quality standards subcommittee of the American Academy of Neurology. Neurology (2007) 69(1):91–102. 10.1212/01.wnl.0000265517.66976.28 17522387

[14] Truelsen T Beggs S Mathers CD The Global Burden of Cerebrovascular Disease. Geneva, Switzerland: WHO (2006). Available from: http://www.who.int/healthinfo/statistics/bod_cerebrovasculardiseasestroke.pdf

[15] Hanny PE Hauselmann HJ Die Lyme-Krankenheit aus der Sicht des neurologen. Schweiz Med Wochenschr (1987) 117:901–15. 3616582

[16] Weder B Wiedersheim P Matter L Steck A Otto F. Chronic progressive neurological involvement in *Borrelia burgdorferi* infection. J Neurol (1987) 234:40–3. 10.1007/BF00314008 3819785

[17] Kohler J Kern U Kasper J Rhese-Küpper B Thoden U. Chronic central nervous system involvement in Lyme borreliosis. Neurology (1988) 38:863–7. 10.1212/WNL.38.6.863 3368066

[18] Veenendaal-Hilbers JA Perquin WV Hoogland PH Doornbos L. Basal meningovasculitis and occlusion of the basilar artery in two cases of *Borrelia burgdorferi* infection. Neurology (1988) 38:1317–9. 10.1212/WNL.38.8.1317 3399082

[19] Schmutzhard E Pohl P Stockhammer G Kleedorfer B Stanek G Unusual neurological manifestations of second-stage Lyme borreliosis. Ann N Y Acad Sci (1988) 539:495–6. 10.1111/j.1749-6632.1988.tb31919.x

[20] Merlo A Weder B Ketz E Matter L. Locked-in state in *Borrelia burgdorferi* meningitis. J Neurol (1989) 236:305–6. 10.1007/BF00314463 2760649

[21] Lock G Berger G Gröbe H Neuroborreliosis: progressive encephalomyelitis with cerebral vasculitis. Monatsschr Kinderheilkd (1989) 137(2):101–4. 2716736

[22] Olsson JE Zbornikova V. Neuroborreliosis simulating a progressive stroke. Acta Neurol Scand (1990) 81:471–4. 10.1111/j.1600-0404.1990.tb00998.x 2375251

[23] May EF Jabbari B. Stroke in neuroborreliosis. Stroke (1990) 21:1232–5. 10.1161/01.STR.21.8.1232 2202096

[24] Miklossy J Kuntzer T Bogousslavsky J Regli F Janzer RC Meningovascular form of neuroborreliosis: similarities between neuropathological findings in a case of Lyme disease and those occurring in tertiary neurosyphilis. Acta Neuropathol (1990) 80:568–72. 10.1007/BF00294622 2251916

[25] Brogan GX Homan CS Viccellio P. The enlarging clinical spectrum of Lyme disease: Lyme cerebral vasculitis, a new disease entity. Ann Emerg Med (1990) 19:572–6. 10.1016/S0196-0644(05)83017-3 2331105

[26] Kuntzer T Bogousslavsky J Miklossy J Steck AJ Janzer R Regli F. *Borrelia* rhombencephalomyelopathy. Arch Neurol (1991) 48:832–6. 10.1001/archneur.1991.00530200072021 1898257

[27] Hammers-Berggren S Gröndahl A Karlsson M von Arbin M Carlsson A Stiernstedt G. Screening for neuroborreliosis in patients with stroke. Stroke (1993) 24:1393–6. 10.1161/01.STR.24.9.1393 8362437

[28] Reik L Jr. Stroke due to Lyme disease. Neurology (1993) 43:2705–7. 10.1212/WNL.43.12.2705 8255484

[29] Defer G Levy R Brugiéres P Postic D Degos JD. Lyme disease presenting as a stroke in the vertebrobasilar territory: MRI. Neuroradiology (1993) 35:529–31. 10.1007/BF00588714 8232882

[30] Demaerel P Wilms G Casteels K Casaer P Silberstein J Baert AL. Childhood neuroborreliosis: clinicoradiological correlation. Neuroradiology (1995) 37:578–81. 10.1007/BF00593726 8570060

[31] Oksi J Kalimo H Marttila RJ Marjamäki M Sonninen P Nikoskelainen J Inflammatory brain changes in Lyme borreliosis. A report on three patients and review of literature. Brain (1996) 119:2143–54. 10.1093/brain/119.6.2143 9010017

[32] Druschky A Erbguth F Claus D Huk W Neundöfer B Borrelien – Meningoenzephalitis und borrelieninduzierte zerebrale Vaskulitis – Zwei Fallberichte. Akt Neurol (1996) 23:116–9. 10.1055/s-2007-1017844

[33] Keil R Baron R Kaiser R Deuschl G Vasculitis course of neuroborreliosis with thalamic infarct. Nervenarzt (1997) 68:339–41. 10.1007/s001150050133 9273464

[34] Schmitt AB Kuker W Nacimiento W Neuroborreliose mit ausgeprägter zerebraler Vaskulitis und multiplen Hirninfarkten. Nervenarzt (1999) 70:167–71. 10.1007/s001150050418 10098153

[35] Laroche C Lienhardt A Boulesteix J Ischemic stroke caused by neuroborreliosis. Arch Pediatr (1999) 6:1302–5. 10.1016/S0929-693X(00)88893-4 10627902

[36] Deloizy M Devos P Stekelorom T Testard D Belhadia A Left sided sudden hemiparesis linked to a central form of Lyme disease. Rev Neurol (2000) 156:1154–6. 11139733

[37] Wilke M Eiffert H Christen HJ Hanefeld F. Primarily chronic and cerebrovascular course of Lyme neuroborreliosis: case reports and literature review. Arch Dis Child (2000) 83:67–71. 10.1136/adc.83.1.67 10869004 PMC1718399

[38] Klingebiel R Benndorf G Schmitt M von Moers A Lehmann R. Large cerebral vessel occlusive disease in Lyme neuroborreliosis. Neuropediatrics (2002) 33:37–40. 10.1055/s-2002-23589 11930275

[39] Heinrich A Khaw AV Ahrens N Kirsch M Dressel A Cerebral vasculitis as the only manifestation of *Borrelia burgdorferi* infection in a 17-year-old patient with basal ganglia infarction. Eur Neurol (2003) 50:109–12. 10.1159/000072510 12944718

[40] Schmiedel J Gahn G von Kummer R Reichmann H Cerebral vasculitis with multiple infarcts caused by Lyme disease. Cerebrovasc Dis (2004) 17:79–81. 10.1159/000073904 14534380

[41] Romi F Kråkenes J Aarli JA Tysnes OB Neuroborreliosis with vasculitis causing stroke-like manifestations. Eur Neurol (2004) 51:49–50. 10.1159/000075090 14639033

[42] Cox MG Wolfs TF Lo TH Kappelle LJ Braun KP. Neuroborreliosis causing focal cerebral arteriopathy in a child. Neuropediatrics (2005) 36:104–7. 10.1055/s-2005-837573 15822023

[43] Jacobi C Schwark C Kress B Hug A Storch-Hagenlocher B Schwaninger M. Subarachnoid hemorrhage due to *Borrelia burgdorferi*-associated vasculitis. Eur J Neurol (2006) 13:536–8. 10.1111/j.1468-1331.2006.01335.x 16722982

[44] Habek M Mubrin Z Brinar VV. Avellis syndrome due to borreliosis. Eur J Neurol (2007) 14:112–4. 10.1111/j.1468-1331.2006.01528.x 17222124

[45] Topakian R Stieglbauer K Nussbaumer K Aichner FT. Cerebral vasculitis and stroke in Lyme neuroborreliosis. Two case reports and review of current knowledge. Cerebrovasc Dis (2008) 26:455–61. 10.1159/000155982 18810231

[46] Van Snick S Duprez TP Kabamba B Van De Wyngaert FA Sindic CJ. Acute ischaemic pontine stroke revealing Lyme neuroborreliosis in a young adult. Acta Neurol Belg (2008) 108:103–6. 19115674

[47] Rénard C Marignier S Gillet Y Roure-Sobas C Guibaud L Des Portes V Acute hemiparesis revealing a neuroborreliosis in a child. Arch Pediatr (2008) 15:41–4. 10.1016/j.arcped.2007.10.008 18155890

[48] Sparsa L Blanc F Lauer V Cretin B Marescaux C Wolff V Recurrent ischemic strokes revealing Lyme meningovascularitis. Rev Neurol (2009) 165:273–7. 10.1016/j.neurol.2008.06.010 18760428

[49] Janmaat M Gravendeel JP Uyttenboogaart M Vroomen PC Brouwer OF Luijckx GJ. Local intra-arterial thrombolysis in a 4-year-old male with vertebrobasilar artery thrombosis. Dev Med Child Neurol (2009) 51:155–8. 10.1111/j.1469-8749.2008.03232.x 19191847

[50] Katchanov J Siebert E Klingebiel R Endres M. Infectious vasculopathy of intracranial large- and medium-sized vessels in neurological intensive care unit: a clinico-radiological study. Neurocrit Care (2010) 12:369–74. 10.1007/s12028-010-9335-4 20146025

[51] Buchwald F Abul-Kasim K Tham J Hansen BU Fatal course of cerebral vasculitis induced by neuroborreliosis. Neurol India (2010) 58:139–41. 10.4103/0028-3886.60407 20228487

[52] Rey V Du Pasquier R Muehl A Péter O Michel P Multiple ischemic strokes due to *Borrelia garinii* meningovasculitis. Rev Neurol (2010) 166:931–4. 10.1016/j.neurol.2010.03.010 20434741

[53] Feuchtinger J Lerch-Riedl T Rohringer E Brenneis C Neuroborreliosis in a patient with presumed cryptogenic ischaemic strokes. Akt Neurol (2011) 38:442–3.

[54] Bremell D Säll C Gisslén M Hagberg L. Lyme neuroborreliosis in HIV-1 positive men successfully treated with oral doxycycline: a case series and literature review. J Med Case Rep (2011) 5:465. 10.1186/1752-1947-5-465 21929779 PMC3183041

[55] Atroun T Varvat J Exbrayat S Cazorlab C Carricajo A Epinat M TIA revealing Lyme neuroborreliosis. Prat Neurol (2013) 4:28–31. 10.1016/j.praneu.2012.12.003

[56] Lebas A Toulgoat F Saliou G Husson B Tardieu M. Stroke due to Lyme neuroborreliosis: changes in vessel wall contrast enhancement. J Neuroimaging (2012) 22:210–2. 10.1111/j.1552-6569.2010.00550.x 21122000

[57] Back T Grünig S Winter Y Bodechtel U Guthke K Khati D Neuroborreliosis-associated cerebral vasculitis: long-term outcome and health-related quality of life. J Neurol (2013) 260:1569–75. 10.1007/s00415-013-6831-4 23329377

[58] Kohns M Karenfort M Schaper J Laws HJ Mayatepek E Distelmaier F Transient ischaemic attack in a 5-year-old girl due to focal vasculitis in neuroborreliosis. Cerebrovasc Dis (2013) 35:184–5. 10.1159/000346597 23429243

[59] Kurian M Pereira VM Vargas MI Fluss J. Stroke-like phenomena revealing multifocal cerebral vasculitis in pediatric Lyme neuroborreliosis. J Child Neurol (2015) 30:1226–9. 10.1177/0883073814552104 25316727

[60] Karadag YS Bilen S Mühürdaroğlu M Oztekin N Hatiboğlu G Ak F Neuroborreliosis in Turkey. Research (2014) 1:831 10.13070/rs.en.1.831

[61] Juric S Janculjak D Tomic S Butkovic Soldo S Bilic E Epileptic seizure as initial and only manifestation of neuroborreliosis: case report. Neurol Sci (2014) 35:793–4. 10.1007/s10072-014-1648-1 24481715

[62] Zajkowska J Garkowski A Moniuszko A Czupryna P Ptaszyńska-Sarosiek I Tarasów E Vasculitis and stroke due to Lyme neuroborreliosis – a review. Infect Dis (Lond) (2015) 47:1–6. 10.3109/00365548.2014.961544 25342573

[63] Wittwer B Pelletier S Ducrocq X Maillard L Mione G Richard S. Cerebrovascular events in Lyme neuroborreliosis. J Stroke Cerebrovasc Dis (2015) 24:1671–8. 10.1016/j.jstrokecerebrovasdis.2015.03.056 26002071

[64] Li S Vytopil M Hreib K Craven DE. Lyme disease presenting as multiple ischaemic strokes. Pract Neurol (2015) 15:284–8. 10.1136/practneurol-2014-001072 25882056

[65] Lenherr N Walther K Schneider J Woerner A Hess M Neuroborreliosis-associated verebral vasculitis. An uncommon manifestation of Lyme disease in a 9-year-old boy. Glob Pediatr Health (2015) 2:2333794X15601840 10.1177/2333794X15601840 PMC4784641 27335977

[66] Blažina K Miletić V Relja M Bažadona D. Cerebral sinuvenous thrombosis: a rare complication of Lyme neuroborreliosis. Wien Klin Wochenschr (2015) 127:65–7. 10.1007/s00508-014-0622-5 25341456

[67] Almoussa M Goertzen A Fauser B Zimmermann CW. Stroke as an unusual first presentation of Lyme disease. Case Rep Neurol Med (2015) 2015:389081. 10.1155/2015/389081 26788385 PMC4695636

[68] Allen NM Jungbluth H Lyme neuroborreliosis: a potentially preventable cause of stroke. J Pediatr (2016) 170:334–e1. 10.1016/j.jpeds.2015.11.077 26775545

[69] Huys AC Lalive PH Haller S Meningoencephalitis with microinfarcts in early neuroborreliosis. Neuroradiology (2016) 58(5):533–4. 10.1007/s00234-016-1657-2 26861491

[70] Back T Grünig S Neuroborreliosis-associated cerebral vasculitis – an update. Neurol Psychiatry Brain Res (2016) 22(3–4):162–6. 10.1016/j.npbr.2016.09.002

[71] Zhang Y Lafontant G Bonner FJ Jr. Lyme neuroborreliosis mimics stroke: a case report. Arch Phys Med Rehabil (2000) 81:519–21. 10.1053/mr.2000.4431 10768546

[72] Lindgren E Jaenson TG Lyme Borreliosis in Europe: Influences of Climate and Climate Change, Epidemiology, Ecology and Adaptation Measures. (2006). Available from: http://www.euro.who.int/__data/assets/pdf_file/0006/96819/E89522.pdf

[73] European Registers of Stroke (EROS) Investigators Heuschmann PU Di Carlo A Bejot Y Rastenyte D Ryglewicz D Incidence of stroke in Europe at the beginning of the 21st century. Stroke (2009) 40:1557–63. 10.1161/STROKEAHA.108.535088 19325154

[74] Oschmann P Dorndorf W Hornig C Schäfer C Wellensiek HJ Pflughaupt KW. Stages and syndromes of neuroborreliosis. J Neurol (1998) 245:262–72. 10.1007/s004150050216 9617706

[75] Alba MA Espígol-Frigolé G Prieto-González S Tavera-Bahillo I García-Martínez A Butjosa M Central nervous system vasculitis: still more questions than answers. Curr Neuropharmacol (2011) 9:437–48. 10.2174/157015911796557920 22379458 PMC3151598

[76] Visoná de Figueiredo NS Moraes Angst DB Mendes LS Machado MF Guimarães Rocha MS Dozzi Brucki SM Basilar artery aneurysm in a woman with syphilis. JAMA Neurol (2015) 72:720–1. 10.1001/jamaneurol.2015.0154 25915828

[77] Asdaghi N Muayqil T Scozzafava J Jassal R Saqqur M Jeerakathil TJ The re-emergence in Canada of meningovascular syphilis: 2 patients with headache and stroke. CMAJ (2007) 176:1699–700. 10.1503/cmaj.070371 17548381 PMC1877856

[78] Hinterseher I Gäbel G Corvinus F Lück C Saeger HD Bergert H Presence of *Borrelia burgdorferi* sensu lato antibodies in the serum of patients with abdominal aortic aneurysms. Eur J Clin Microbiol Infect Dis (2012) 31:781–9. 10.1007/s10096-011-1375-y 21842293 PMC3319877

[79] Djukic M Schmidt-Samoa C Lange P Spreer A Neubieser K Eiffert H Cerebrospinal fluid findings in adults with acute Lyme neuroborreliosis. J Neurol (2012) 259:630–6. 10.1007/s00415-011-6221-8 21898139 PMC3319903

[80] Lakos A. CSF findings in Lyme meningitis. J Infect (1992) 25:155–61. 10.1016/0163-4453(92)93966-T 1431169

[81] Bremell D Dotevall L. Oral doxycycline for Lyme neuroborreliosis with symptoms of encephalitis, myelitis, vasculitis or intracranial hypertension. Eur J Neurol (2014) 21:1162–7. 10.1111/ene.12420 24684211

