# Supplementary material for: Cerebrovascular Manifestations of Lyme Neuroborreliosis—A Systematic Review of Published Cases
Source: Front Neurol. 2017 Apr 20;8:146. doi: 10.3389/fneur.2017.00146 (PMC5397664; doi:10.3389/fneur.2017.00146)
Supplement: Supplementary file 1 [file Data_Sheet_1.doc]

**Supplementary web appendix**

Clinical features, CSF cytosis and imaging studies in the previously reported cases cerebrovascular manifestations of Lyme neuroborreliosis

| **Reference** | **Year of publication** | **Case** | **Age (years)/Sex** | **History of tick bite/ EM** | **Signs and symptoms** | **CSF cells/mm^3^** | **CSF protein level (mg/L)** | **Radiological and/or histopathological features** | **Treatment** | **Outcome** |
| --- | --- | --- | --- | --- | --- | --- | --- | --- | --- | --- |
| Uldry et al. | 1987 | 1 | 40/F | Yes/Yes | Recurrent ischaemic events during 3 years following a tick bite. Diplopia, confusion and a stumbling gait. Left ataxic hemiparesis. Blurred vision with numbness in the hands with left-sided hemiparesis. History of headache associated with weakness and paraesthesia around the mouth and in the left hand. | 28 | 2670 | CT: a hypodense area in the L thalamus and the old lesion in the R lenticular nucleus without contrast enhancement. DSA: segmental narrowing and obstruction of branches of MCA and ACA on both sides and narrowing of the thalamic arteries | penicillin G, steroids | complete recovery |
| [Midgard](http://www.ncbi.nlm.nih.gov/pubmed?term=Midgard R%5BAuthor%5D&cauthor=true&cauthor_uid=3036051) and Hofstad | 1987 | 2 | 49/F | Yes/n.a. | Acute transient dysphasia and muscular weakness in both shoulders. Peripheral facial nerve palsy. History of radiculitis and headache. | 54 | 940 | CT: infarction in the L capsula interna. Cerebral angiography: multiple stenoses of small and large intracerebral arteries on both sides | penicillin G | complete recovery |
| Hänny and Häuselmann | 1987 | 3 | 70/F | Yes/No | Right sided hemiparesis. History of facial nerve palsy | 140 | n.a. | CT and MRI: multiple perivascular white matter lesions | penicillin G | complete recovery |
|  |  | 4 | 74/F | n.a./n.a. | Left-sided hemiparesis, hemianopsia, dysphagia, vertigo and disturbances in consciousness. History of facial and trochlear nerve palsy | 114 | n.a. | CT: R occipital infarct, and bilateral cerebellar infarcts | n.a. | n.a. |
| Weder et al. | 1987 | 5 | 23/M | n.a./n.a. | Hemiparesis. History of meningitis and cranial neuritis | 1125 | 6400 | CT: hypodense lesion in the L temporoparietal area with enhancement of kontrast medium | penicillin G | complete recovery |
|  |  | 6 | 61/F | n.a./n.a. | Hemiparesis. History of meningitis and cranial neuritis | 136 | 5140 | CT: multiple hypodensities compatible with a multi-infarct syndrome | penicillin G | complete recovery |
| Kohler et al. | 1988 | 7 | 20/M | n.a./n.a. | Left-sided transient hemiparesis, severe headache and adynamia. Two months later the patient experienced right-sided hemiparesis and was confused. | 30 | 2940 | CT and MRI: bilateral thalamic infarcts | penicillin G, steroids | incomplete recovery with neurological impairment |
|  |  | 8 | 36/M | n.a./n.a. | Recurrent right-sided hemiparesis, aphasia and severe headache. | 550 | 835 | CT: two hypodense areas in the white matter, right-sided frontal and left-sided parietal. MRI: multiple hyperintense lesions in the periventricular area and in the white matter of both hemispheres. TCD: stenosis of intracranial part of the L ICA | penicillin G | complete recovery |
| Veenendaal-Hilbers et al. | 1988 | 9 | 27/F | No/No | Recurrent episodes of acute loss of strength in the left arm and a left-sided facial palsy. History of continuous headaches radiating towards the neck for 3 months. Slight bilateral papilledema. | 250 | 2000 | CT: no abnormalities. DSA: occlusion of the BA and stenoses in the L VA | penicillin G, steroids | complete recovery |
|  |  | 10 | 24/M | Yes/n.a. | Vertigo and slurred speech. Difficulty with walking, climbing stairs and urinary incontinence during 3 months. Bilateral Babinski signs. | 33 | 1420 | CT: no abnormalities. DSA: occlusion of the BA and irregular pat tern in the vascular wall near the junction of the R VA and BA; the abnormality extender toward the proximal part of the anterior inferior cerebellar artery | penicillin G | n.a. |
| Schmutzhard et al. | 1988 | 11 | 44/F | n.a./n.a. | Left-sided hemiparesis. | 130 | n.a. | CT and MRI: R parietal infarction. Cerebral angiography - no abnormalities (performed 10 days after the onset of illness) | penicillin G | incomplete recovery with neurological impairment |
| Merlo et al. | 1989 | 12 | 28/F | No/Yes | Locked-in syndrome. Facial and hypoglossal nerve palsy. Respiratory insufficiency requiring intubation. History of radicular pain, fatigue, weight loss, headache and nausea with vomiting. | 180 | 2100 | CT and MRI: no abnormalities | penicillin G | incomplete recovery with neurological impairment |
| Lock et al. | 1989 | 13 | 16/M | No/n.a. | Left-sided hemiparesis, ataxia and left-sided facial nerve palsy. History of emotional lability | 644 | n.a. | CT and MRI: multiple bilaterally situated ischemic lesions with contrast enhancement. TCD: stenosis of R MCA | penicillin G | complete recovery |
| Olsson and Zbornikova | 1990 | 14 | 55/M | Yes/Yes | Left-sided hemiparesis and left-sided central facial palsy. History of fatigue and depression. | 30 | 2960 | CT: no abnormalities | penicillin G | complete recovery |
| May and Jabbari | 1990 | 15 | 20/M | n.a./No | Acute light-headedness, vertical diplopia, and dysarthria. Marked impairment of short-term memory. Right and left central facial nerve palsy and mild clumsiness of the right upper ex termity. Intermittent occipital headaches | 770 | 2100 | MRI: high-intensity signal in L paramedian thalamus, in distribution of posterior choroidal artery. Enhancing lesion in R anterior thalamus in distribution of tuberothalamic artery | ceftriaxone | complete recovery |
| Brogan et al. | 1990 | 16 | 37/F | No/No | Severe headache for 24 hours with nausea, followed by left ptosis and flattening of the left nasolabial fold and grand mal seizures | 1 | 1780 | CT: acute infarct in the R thalamic region near the posterior limb of the internal capsule. Cerebral angiography: segmental narrowing irregularities and beading of small and moderate size vessels with relative dilation in the segments distal to narrowing | ceftriaxone, steroids | complete recovery |
| [Kuntzer](http://www.ncbi.nlm.nih.gov/pubmed/?term=Kuntzer T%5BAuthor%5D&cauthor=true&cauthor_uid=1898257)  et al. | 1991 | 17 | 50/M | No/No | Right-sided hemiparesis and after 3 days, a superimposed left-sided hemiparesis. History of multiple cranial nerve dysfunction and radiculitis | 212 | 1290 | Autopsy findings: bilateral tonsillar herniation; two small infarcts (one in the tegmentum and the other in the medulla oblongata); obliterative inflammatory vasculopathy of numerous brain vessels); *Borrelia* spirochetes were found by microscopic analysis in the leptomeninges at the level of medulla oblongata and around small subependymal vessels of the ventricles. | No antibiotics were administered; only steroids were used | died (death occurred due to ventilator-associated pneumonia at month 30) |
| Hammers-Berggren et al. | 1993 | 18 | 66/F | n.a./n.a. | Acute transient dysphasia that disappeared within a few hours. History of headache and radicular pain for several months followed by vertigo, balance disturbances, hand tremor, blurred vision, and weight loss. | 74 | 1900 | CT: no abnormalities. | doxycycline | complete recovery |
| Reik L | 1993 | 19 | 56/F | No/Yes | Left facio-brachio-crural hemiparesis, left hemiataxia and left Babinski sign. History of headaches, neck pain and stiffness, and left peripheral facial palsy | 238 | 1740 | CT: low-density lesion in the posterior limb of the R internal capsule. MRI: hyperintense lesion in the R caudate nucleus, globus pallidus and anterior limb of the internal capsule, and several smaller periventricular lesions, and lesion in the posterior limb of the R internal capsule | doxycycline, ceftriaxone, steroids | complete recovery |
| Defer et al. | 1993 | 20 | 28/F | n.a./No | Acute onset of dizziness, an unstable gait and nystagmus followed by aphonia, dysphagia, and tetraparesis. History of headache and intermittent radicular pain for 3 months. | 227 | 960 | CT: no abnormalities. MRI: infarction in the medial part of the pons. DSA: no abnormalites | ceftriaxone | complete recovery |
| Demaerel et al. | 1995 | 21 | 10/F | Yes/No | Acute left-sided hemiparesis, headache and peripheral facial palsy | 34 | 420 | CT: no abnormalities. MRI: multiple bilaterally situated subcortical white matters lesions | ceftriaxone | complete recovery |
| Druschky et al. | 1996 | 22 | 50/M | Yes/No | Right-sided hemiparesis and visual impairment, changes in behavior | 539 | 1230 | CT: multiple bilaterally situated ischemic lesions; cortical atrophy. DSA: DSA- irregularities and narrowing of the R ACA and R MCA | doxycycline, amoxicillin, ceftriaxone, steroids | complete recovery |
|  |  | 23 | 54/F | Yes/No | Left-sided hemiparesis, vertigo and nystagmus. History of headache | 101 | 1100 | CT: no abnormalities. MRI: L internal capsule infarct. MRA: no abnormalities. | doxycycline, ceftriaxone, steroids | complete recovery |
| Oksi et al. | 1996 | 24 | 40/M | No/No | Generalized seizure | n.a. | 688 | CT: no abnormalities. MRI: multiple contrast-enhancing lesions in the L frontal lobe near the meninges. Histological examination of brain tissue samples: lymphocytes in the walls of leptomeningeal and small penetrating arteries as well as in the perivascular space of the latter. The adjacent cortex was slightly oedematous with very mild astrocytic gliosis. A PCR analysis of brain tissue samples detected DNA of *B. burgdorferi.* | ceftriaxone, amoxicillin, azithromycin, doxycycline | complete recovery |
|  |  | 25 | 11/F | n.a./n.a. | Paresis of the right lower limb. History of cccasional episodes of hyperactivity followed by headache for several weeks | n.a. | n.a. | CT and MRI: periventricular low density enhancing lesion located in parietal lobe white matter. Histological examination of the lesion: increased number of small vessels with thickened walls and prominent endothelial cells. Lymphocytes occurred in the walls of some vessels. | ceftriaxone, amoxicillin, probenecid | complete recovery |
| [Keil et al.](http://www.ncbi.nlm.nih.gov/pubmed/?term=Keil R%5BAuthor%5D&cauthor=true&cauthor_uid=9273464) | 1997 | 26 | 20/M | Yes/No | Left-sided hemiparesis | 1500 | 2970 | MRI: R thalamus infarction. DSA: stenosis of the right thalamic vessels. | ceftriaxone | complete recovery |
| Oksi et al. | 1998 | 27 | 42/F | No/Yes | Sudden, severe headache and unconsciousness. History of right sided peripheral facial nerve paresis followed by radicular pain | 5 | 619 | CT: subarachnoid haemorrhage. DSA: an aneurysym arising from the BA near the bifurcation of the left superior cerebellar artery | ceftriaxone, amoxicillin | complete recovery |
| Schmitt et al. | 1999 | 28 | 50/F | n.a./n.a. | Right-sided hemiparesis, hemianopsia and reduced consciousness. History of headache | 68 | 1400 | CT: no abnormalities. MRI: multiple cerebral infarctions; leptomeningeal enhancement on T1 images. DSA: stenosis of BA, R PCA and R cerebellar artery. TCD: stenosis of BA | ceftriaxone, steroids, cyclophosphamide | incomplete recovery with neurological impairment |
| Laroche et al. | 1999 | 29 | 9/M | No/Yes | Left-sided hemiparesis | 65 | n.a. | CT and MRI: R ischemic lenticular lesion | ceftriaxone | complete recovery |
| Polet and Weinstein | 1999 | 30 | 33/M | n.a./n.a. | Left-sided hemiparesis and headache; right facial nerve palsy | 246 | 3490 | CT and MRI: L anterior infarction. DSA: narrowing of the L ACA and an aneurysm of the BA | ceftriaxone | complete recovery |
| Deloizy et al. | 2000 | 31 | 27/M | n.a./n.a. | Left-sided hemiparesis | lymphocytic pleocytosis | n.a. | CT and MRI: R capsulo-thalamic infarction | n.a. | complete recovery |
| Wilke et al. | 2000 | 32 | 15/F | Yes/No | Left-sided hemiparesis and right-sided atalia. A history of abdominal discomfort, vomiting, headache, and slight mental slowing for several weeks. | 64 | 1132 | MRI: lesion in the L basal ganglia and the posterior internal capsulee | penicillin G, ceftriaxone, steroids | complete recovery |
| Seijo Martínez et al. | 2001 | 33 | 48/M | n.a./n.a. | Acute headache, confusion, severe left-sided hemiparesis with sensory deficit and homonymous hemianopsia. Progressive spastic paraparesis of months' duration. | 10 | 592 | CT: extensive R temporal lobe hemorrhage with subarachnoid invasion. Brain angiographic and MRA excluded hemorrhage - predisposing vascular abnormalities. | ceftriaxone | incomplete recovery with neurological impairment |
| Klingebiel et al. | 2002 | 34 | 12/F | No/No | Right-sided hemiparesis. History of slowly increasing fatigue over a period of 3 months, transient headache with nausea and pain in the right lower extermity | 45 | 7500 | MRI: hyperintense lesions in the L frontoparietal region and within the ipsilateral basal ganglia on T_2_ images. MRA and DSA: stenosis of the terminal L ICA and L ACA and L MCA. | ceftriaxone, cefotaxime | complete recovery |
| Ansari et al. | 2002 | 35 | 6/F | No/No | Headaches for 3 weeks and diplopia for 2 days. History of emesis 3 weeks prior to examination. Mild bilateral disc edema | 20 | n.a. | MRI: no abnormalities. MR venography: complete occlusion of R and partial occlusion of the L transverse sinus, consistent with venous sinus thrombosis | ceftriaxone | complete recovery |
| Scheid et al. | 2003 | 36 | 56/F | Yes/n.a. | Subacute attacks of strong headaches and left-sided abducens nerve palsy. History of peripheral facial palsy and migratory arthralgias | n.a. | n.a. | CT: right-sided atypical temporoparietal haemorrhage extending into the subarachnoid space. DSA: no abnormalities. | n.a. | n.a. |
| Heinrich et al. | 2003 | 37 | 17/F | No/No | Left brachiofacial hemiparesis, aphasia and right-sided hemianopsia. | 73 | n.a. | MRI: ischemic lesion in the R lentiform and caudate nucleus. MRA: stenosis in the R MCA and irregular pat tern of other intracranial vessels. TCD: high-grade stenosis of the R MCA and moderate stenosis of the R ACA and PCA. | ceftriaxone, steroids | complete recovery |
| Schmiedel  et al. | 2004 | 38 | 38/F | Yes/No | Acute onset of left-sided hemiparesis, somnolence and disorientation and hallucinations. History of headaches, dorsalgia and neck pain. Lef-side facial nerve palsy | 298 | 7458 | CT: infarction of the R lentiform nucleus and internal capsule. MRI: ischemic lesion of the R basal ganglia and the temporal cortex. Meningobasal enhancement of both hemisperes. MRA: high-grade stenosis of the R ICA. TCD: increased blood flow velocities in both MCA and ACA. | ceftriaxone, steroids | incomplete recovery with neurological impairment |
| Romi et al. | 2004 | 39 | 56/M | No/No | Right-sided hemiparesis. History of headache and neck pain followed by bilateral peripheral facial nerve paresis. | 250 | 1800 | MRI: hiperintense lesion in the medulla oblongata and multiple hyperintense lesions in subcortical areas and deeper white matter. TCD: no abnormalities | ceftriaxone, steroids | complete recovery |
| Cox et al. | 2005 | 40 | 9/F | No/No | Acute right-sided hemiparesis with central right-sided facial nerve paresis and aphasia. History of unclear episode of dysartria 2 weeks before presentation. Several non-specific complaints during the 1.5 years preceding cerebral infarct, such as sleeplessness, easy fatigue, dizziness, and unaccountable pain in arms and legs | 13 | 720 | MRI: infarction in the head of the caudate nucleus, lentiform nucleus, and corona radiata in the L hemisphere, and a possibile of small lacunar infarct in the R thalamus. MRA and DSA: regular segmental stenosis of the L MCA and either an occlusion or a congenital hypoplasia of the L ACA. | ceftriaxone | complete recovery |
| Jacobi et al. | 2006 | 41 | 58/F | Yes/Yes | Acute headache and nuchal rigidity. History of radicular pain | 1067 | n.a. | CT: occipital subarachnoid haemorrhage. CTA: no abnormalities. DSA: irregularities in the R PCA | cefuroxime, steroids | complete recovery |
| Habek et al. | 2007 | 42 | 67/M | No/Yes | Avellis' syndrome. History of peripheral facial palsy | 6 | 270 | CT: no abnormalities. MRI: high signal intensity in the R medulla oblongata. MRA: no abnormalities | ceftriaxone | complete recovery |
| Topakian et al. | 2008 | 43 | 46/M | Yes/No | Right-sided hemiplegia and aphasia. History of headache starting several weeks before admission. | 308 | 393 | MRI: ischemic infarction in the territory of the L MCA and perimedullary pial enhancement on T_1_ images. MRA: multiple stenotic vessels of the circle of Willis with marked reduction of flow in the distal L ICA and occlusion of the L MCA, diameter irregularities in the R MCA and PCA. TCD: increased velocities in the ACA and MCA | ceftriaxone | complete recovery |
|  |  | 44 | 37/M | No/No | Disorientation to time and place and major neuropsychological deficits with receptive dysphasia. History of malaise, fatigue and headaches accompanied by nausea and vomiting several months before admission. | 138 | 186 | MRI: infarctions in the L basal ganglia and temportal and occipital cortex. Perimedullary pial enhancement on T_1_ images. MRA and DSA: irregularity and a reduction in the caliber of the L ICA and MCA. | ceftriaxone | complete recovery |
| Van Snick et al. | 2008 | 45 | 23/M | Yes/No | Ataxic gait, right kinetic hemiataxia, homolateral hemiparesis and dysarthria. History of backache and headache after tick bite. | 160 | 1370 | MRI: bilateral abnormally hyperintense foci within the pons with a smaller R parasagittal lesion and a larger L parasagittal one. MRA: no abnormalities | ceftriaxone | complete recovery |
| Rénard et al. | 2008 | 46 | 11/M | No/No | Recurrent episodes of right-sided hemiparesis and aphasia. History of headache and fatigue since 1 year. | 450 | 1800 | MRI: L capsulothalamic lesion. MRA: narrowing of the BA and L MCA. | ceftriaxone | complete recovery |
| Sparsa et al. | 2009 | 47 | 64/M | n.a./n.a | Right-sided hemiparesis followed by cognitive impairment and a bilateral cerebellar syndrome, blurred vision. History of headache and arthralgia | 122 | 4550 | CT: L thalamic hypodensity on the initial cerebral CT scan. MRI: R hyperintense thalamic and pedoncular lesions and some R cerebellar, L linear thalamic and paraventricular. MRA: multiple intracranial stenoses | ceftriaxone, steroids | complete recovery |
| Janmaat et al. | 2009 | 48 | 4/M | n.a./n.a. | Aphasia, atalia and tetraparesis | n.a. | n.a. | MRI: hyperintense lesions in the pons and caudal part of the mesencephalon. MRA: complete occlusion of the BA with exception of the distal part and thrombosis of the distal part of the L VA | n.a. | complete recovery |
| Katchanov et al. | 2010 | 49 | 56/F | n.a./n.a. | Locked in syndrome | 93 | n.a. | MRI: multiple ischemic lesions in the bilateral medulla oblongata, pons and R PCA territory; leptomeningeal enhancement on T_1_ images. CTA: occlusion of both VAs in the V4-segment | ceftriaxone, ampicillin steroids, | incomplete recovery with neurological impairment |
| Buchwald et al. | 2010 | 50 | 25/M | Yes/No | Sudden onset of right-sided hemiparesis, and subsequent dysphasia and paresis of the left leg several weeks later | 6 | 1880 | CT and MRI: infarction in the territory of the L ACA and small L lacunar subcortical infarction in the white matter of the putamen and adjacent parts of the L internal capsule. MRA: extensive changes in the calibre of BA, both MCAs and occlusion of the L ACA. | doxycycline, steroids, cyclophosphamide | died (3 weeks after the last hospitalization) |
| Rey et al. | 2010 | 51 | 51/M | Yes/Yes | Left-sided hemiparesis, ataxia, hemianopsia and disturbances in consciousness. History of malaise and cognitive impairment for several months | 68 | 1419 | CT: R internal capsule infarction. MRI: multiple ischemic lesions - R lentriculostriatal, R and L occipital. MRA: multiple stenosis on ACA on both sides, MCA on both sides and carotid ending. | ceftriaxone, steroids, cyclophosphamide | incomplete recovery with neurological impairment |
| Adamaszek et al. | 2010 | 52 | 29/M | No/No | Malaise, pain around the neck, headache and fever. | 297 | 692 | MRI: bright signal on T1- and T2-weighted images in the superior sagittal sinus and R transverse sinus corresponding to lack of flow on MR venography, consistent with subacute cerebral venous thrombosis. Contrast images showed meningeal enhancement | ceftriaxone, ampicillin | complete recovery |
| Feuchtinger et al. | 2011 | 53 | 55/F | No/No | Right-sided hemiparesis. History of fatigue for the last two months | 79 | 472 | CT and MRI: infarctions in the L ventral medulla oblongata and R thalamus. MRA and TCD: no abnormalities | ceftriaxone | complete recovery |
| Bremell et al. | 2010 | 54 | 39/M | n.a./n.a | Sudden onset of vertigo and hearing loss .History of slowly increasing headaches, weakness in both legs and right hand tremor | 492 | 2860 | MRI: a pontine infarction. | doxycycline | incomplete recovery with neurological impairment |
| Atroun et al. | 2013 | 55 | 64/M | Yes/n.a. | Recurrent transient aphasia, vertigo. | 53 | 2350 | CT and MRI: no abnormalities. MRA: irregular pattern of the L MCA | ceftriaxone | complete recovery |
| Lebas et al. | 2012 | 56 | 8/M | n.a./Yes | Right-sided hemiparesis, somnolence and vomiting | 19 | 540 | MRI: hyperintense lesion on the paramedial part of the L pons and hyperintense lesion in the L cerebellum with restricted diffusion. Focal enhancement of the arterial wall of the BA. MRA: last third of the BA was irregular. | ceftriaxone, steroids | complete recovery |
| Back et al. | 2013 | 57 | 47/F | Yes// | n.a. | 18 | 2181 | MRI: multiple lesions – L precentral, R parietal, R centrum semiovale. DSA: generalized intracranial vasospasm | all patients from these study were treated with ceftriaxone; four of them received steroids | incomplete recovery with neurological impairment |
|  |  | 58 | 49/F | Yes/ | n.a. | 76 | 4443 | MRI: multiple lesions – bilateral pontine and R cerebellar. MRA: BA stenosis, later occlusion |  | incomplete recovery with neurological impairment |
|  |  | 59 | 69/M | Yes/ | n.a. | 71 | 962 | MRI: multiple lesions – bilateral cerebellar, pontine, bilateral thalamic, midbrain and both PCA territories. MRA: BA thrombosis |  | died (in the acute stage of the disease) |
|  |  | 60 | 73/M | Yes/ | n.a. | 49 | 876 | MRI: L hemispheric lesion. MRA: no abnormalities |  | complete recovery |
|  |  | 61 | 66/F | Yes/ | n.a. | 307 | 1419 | MRI: multiple lesions – L MCA and PCA territories. MRA: irregular VAs. TCD: L PCA stenosis |  | incomplete recovery with neurological impairment |
|  |  | 62 | 55/M | Yes/ | n.a. | 2 | 2337 | MRI: miltiple lesions – R MCA territory. MRA: multiple stenosis of R MCA and ACA |  | complete recovery |
|  |  | 63 | 77/F | Yes/ | n.a. | 42 | 169 | MRA: L subcortical lesion. MRA: no abnormalities. MR venography: sagittal sinus thrombosis |  | incomplete recovery with neurological impairment |
|  |  | 64 | 74/F | Yes/ | n.a. | 530 | 2138 | MRI: multiple lesions – R striatum, L cerebellar, R pontine. MRA: irregular VAs and R PCA. MRI: contrast enhancement of brainstem |  | incomplete recovery with neurological impairment |
|  |  | 65 | 70/M | Yes/ | n.a. | 31 | 982 | MRI: multiple lesions – R caudate, R subcortical, L thalamus. MRA: no abnormalities. DSA: R ICA stenosis |  | complete recovery |
|  |  | 66 | 52/M | Yes/ | n.a. | 295 | 4479 | MRI: no abnormalities. MRA: high grade stenosis of top of the BA |  | complete recovery |
|  |  | 67 | 46/M | Yes/ | n.a. | 331 | 2294 | MRI: lesions in R thalamus and peduncle. MRA, TCD and DSA: no abnormalities. |  | complete recovery |
| Kohns et al. | 2013 | 68 | 5/F | Yes/No | Transient right-sided hemiparesis. History of recurrent short episodes of vertigo. | 25 | n.a. | MRI: an altered signal in the posterior part of the L putamenm and in a L periventricular area. MRA: several short stenoses at the branching site of the L MCA | ceftriaxone, steroids | complete recovery |
| Kurian et al. | 2015 | 69 | 12/M | Yes/No | Sudden onset of severe headache, left central facial paresis, left-sided hemiparesis, unsteadiness and dysarthria. History of nausea, vomiting nd headache for several months | 1152 | 4500 | CT: no parenchymal changes. MRI: no parenchymal lesions; contrast enhancement of the vessel wall of the BA on T_1_ images. MRA: multiple arterial narrowing of the R and L ACA, L MCA and BA. | ceftriaxone, steroids | complete recovery |
| Karadag et al. | 2014 | 70 | 39/F | Yes/No | Right-sided hemiparesis progressing to tetraparesis and coma in a few hours. History of fatigue and headache for the last one month | 2200 | n.a. | CT: diffuse brain oedema. MRI: hyperintense lesions at frontal, occipital and parietal lobes. MRA: no abnormalities | ceftriaxone, steroids | died (the 10th day of hospitalization |
| Juric et al. | 2014 | 71 | 46/M | Yes/Yes | Transient right-sided hemiparesis with dysphasia; partial motor epileptic seizure with secondary generalization, preceded by general weakness for several days before. | n.a. | n.a. | CT: no abnormalities. MRI: L temporal hyperintense lesion | ceftriaxone | complete recovery |
| Zajkowska et al. | 2015 | 72 | 27/F | Yes/No | Right sided hemiparesis, blurred vision, cerebellar signs | 62 | 920 | MRI: hyperintense lesion in the brainstem. CTA: stenosis of the initial segment of the L ACA and the R PCA | ceftriaxone, steroids | complete recovery |
|  |  | 73 | 56/M | Yes/No. | Recurrent bilateral hemiparesis | 22 | 521 | MRI: multifocal cerebral hyperintensities in both hemispheres | ceftriaxone | complete recovery |
|  |  | 74 | 59/F | No/No | Left-sided hemiparesis. short-term memory loss, spatial orientation disorder, psychomotor impairment. Urinary incontinence | 23 | 218 | MRI: hyperintense lesion in the thalamus and hyperintense areas around the lateral ventricles. MRA: narrowing of the R posterior communicating artery and irregular pattern of intracranial vessels. TCD: low blood flow velocity in both MCAs and in the vertebrobasilar system and high blood flow velocity in the L ACA | ceftriaxone | complete recovery |
|  |  | 75 | 65/F | Yes/No | Recurrent episodes of transient left-sided hemiparesis | 112 | 1059 | CT: no changes. MRI: no changes. | ceftriaxone | complete recovery |
| Wittwer et al. | 2015 | 76 | 66/M | No/n.a. | Recurrent episodes of right- and left-sided hemiparesis. Headaches for several weeks | 42 | 2190 | MRI: L acute anteromedial pontine ischemic lesion and ischemic lesions in the R pons, R lentiform, and caudate nuclei. Enhancement of vessel wall arteries. MRA: L VA occlusion | ceftriaxone, doxycycline | complete recovery |
|  |  | 77 | 64/F | Yes/Yes | Transient left-sided hemiparesis. Gradually worsening daily headaches. Cognitive decline and gait disturbance. | 74 | 770 | CT: no changes. MRI: disseminated lesions, with diffuse meningeal and perivascular contrast enhancement. MRA: no abnormalities | amoxicillin, ceftriaxone | complete recovery |
|  |  | 78 | 71/F | No/n.a. | Progressive gait disturbance, tremor, memory disorder and weight loss. Bilateral pyrami dal syndrome and cerebellar and prioceptive ataxia | 28 | 465 | MRI:disseminated microbleeds, especially in L thalamus, the posteriori fossa, and the cervical spinal cord. MRA: no abnormalities | ceftriaxone | complete recovery |
|  |  | 79 | 39/M | n.a./n.a. | Repetitive episodes of paresis of the right arm and transitory aphasia | <5 | 340 | MRI: several acute ischemic lesions in the R superficial MCA territory. MRA: no abnormalities | ceftriaxone | complete recovery |
|  |  | 80 | 5/F | No/n.a | Vomiting, dysphagia, sensory loss, and nystagmus compatible with a left lateral medullary syndrome. Headaches and nausea for several weeks. | 115 | 1150 | MRI: acute ischemic lesion of the L posterolateral medulla oblongata, and an older R cerebellar infarct. MRA: no abnormalities | ceftriaxone | complete recovery |
| Li et al. | 2015 | 81 | 46/M | No/No | Recurrent left-sided hemiparesis and headache | 100 | 1800 | MRI: R thalamic infarction and infarction in the R paracentral pons. MRA and CTA: stenoses involving bilateral ACAs and the R MCA. | ceftriaxone, penicillin G | incomplete recovery with neurological impairment |
| Lenherr et al. | 2015 | 82 | 9/M | n.a./Yes | Chronic fatigue, headache, intermittent nausea, and vomiting for 2 months. Postural tremor of both hands and horizontal nystagmus | lymphocytosis | n.a. | MRI: infarction of the R cerebellar hemisphere. MRA and DSA: occlusion of the BA | ceftriaxone, steroids | complete recovery |
| Blažina et al. | 2015 | 83 | 73/F | Yes/No | Acute onset of double vision, thunderclap headache, gait instability. Left abducens nerve palsy. Bilateral papilledema on fundoscopic examination. | 44 | 670 | CT and MRI: no abnormalities. TCD: no abnormalities. MR venography: L internal jugular vein thrombosis together with L transversal and sigmoid sinus thrombosis | ceftriaxone, doxycycline | complete recovery |
| Almoussa et al. | 2015 | 84 | 43/M | Yes/ No | Left-sided hemiparesis. Two-week history of malaise, headache and amnestic cognitive impairment | 43 | n.a. | MRI: hyperintense signal in periventricular and in periaqueductal area, in both crura cerebri and in both hypothalami. DWI – R thalamic infarct | ceftriaxone | incomplete recovery with neurological impairment |
| [Huys](http://www.ncbi.nlm.nih.gov/pubmed/?term=Huys AC%5BAuthor%5D&cauthor=true&cauthor_uid=26861491)  et al. | 2016 | 85 | 77/M | n.a/Yes | Acute confusional state, including hallucinations | 37 | 1200 | MRI: hyperintensities in the subarachnoid space and white matter on FLAIR and subtle contrast enhancement on T_1_ images. DWI: restricted diffusion on DWI. MRA and TCD: no abnormalities | ceftriaxone | complete recovery |
| Allen et al. | 2016 | 86 | 15/M | n.a/ Yes | Bilateral facial nerve palsy, right sided limb weakness, cerebellar signs. History of lethargy, anorexia, arthromyalgia and headache. | lymphocytosis | n.a. | MRI: signal abnormality suggesting infarcts in medulla, pons and cerebellum, corresponding with restricted diffision. MRA: Vessel irregularity in the Circle of Willis. FDG-PET: corresponding hypermetabolic areas suggesting inflammation/vasculitis | ceftriaxone | complete recovery |
| Back and Grünig | 2016 | 87 | 49/F | n.a/ n.a | Sudden vomiting, paresthesia of both arms, slurred speech, disturbed coordination of arms and unsteady gait, right-sided hemiparesis A history of fluctuating occipital headache for six months | 76 | 4440 | MRI: meningeal contrast enhancement, L paramedian pontine and R cerebellar infarct. MRA and TCD: high grade stenosis of the BA | ceftriaxone, steroids | incomplete recovery with neurological impairment |
|  |  | 88 | 50/M | n.a/ n.a | Transient disturbance of the visual field and word making trouble. The wife of the patient reported a change in his temper. | 26 | 925 | MRI: infarction in the territory of L MCA. MRA: stenosis of the L MCA | ceftriaxone | complete recovery |

**Abbreviations**

ACA – anterior cerebral artery

BA – basilar artery

CTA - computed tomography angiograpju

DSA - digital subtraction angiography

MCA - middle cerebral artery

MRA – magnetic resonance angiography

MRI: magnetic resonance imaging

n.a. – not available

PCA – posterior cerebral artery

PCR - polymerase chain reaction

VA – vertebral artery

R – right, L – left
